# AfterQC: automatic filtering, trimming, error removing and quality control for fastq data

**DOI:** 10.1186/s12859-017-1469-3

**Published:** 2017-03-14

**Authors:** Shifu Chen, Tanxiao Huang, Yanqing Zhou, Yue Han, Mingyan Xu, Jia Gu

**Affiliations:** 1 Shenzhen Institutes of Advanced Technology, Chinese Academy of Sciences, Xueyuan Road, Shenzhen, China; 2 HaploX BioTechnology, Songpingshan Road, Shenzhen, China; 3 University of Chinese Academy of Sciences, 19 A Yuquan Rd, Shijingshan District, Beijing, China

**Keywords:** NGS, Overlap analysis, Quality control, Data filtering, Bubble

## Abstract

**Background:**

Some applications, especially those clinical applications requiring high accuracy of sequencing data, usually have to face the troubles caused by unavoidable sequencing errors. Several tools have been proposed to profile the sequencing quality, but few of them can quantify or correct the sequencing errors. This unmet requirement motivated us to develop AfterQC, a tool with functions to profile sequencing errors and correct most of them, plus highly automated quality control and data filtering features. Different from most tools, AfterQC analyses the overlapping of paired sequences for pair-end sequencing data. Based on overlapping analysis, AfterQC can detect and cut adapters, and furthermore it gives a novel function to correct wrong bases in the overlapping regions. Another new feature is to detect and visualise sequencing bubbles, which can be commonly found on the flowcell lanes and may raise sequencing errors. Besides normal per cycle quality and base content plotting, AfterQC also provides features like polyX (a long sub-sequence of a same base X) filtering, automatic trimming and K-MER based strand bias profiling.

**Results:**

For each single or pair of FastQ files, AfterQC filters out bad reads, detects and eliminates sequencer’s bubble effects, trims reads at front and tail, detects the sequencing errors and corrects part of them, and finally outputs clean data and generates HTML reports with interactive figures. AfterQC can run in batch mode with multiprocess support, it can run with a single FastQ file, a single pair of FastQ files (for pair-end sequencing), or a folder for all included FastQ files to be processed automatically. Based on overlapping analysis, AfterQC can estimate the sequencing error rate and profile the error transform distribution. The results of our error profiling tests show that the error distribution is highly platform dependent.

**Conclusion:**

Much more than just another new quality control (QC) tool, AfterQC is able to perform quality control, data filtering, error profiling and base correction automatically. Experimental results show that AfterQC can help to eliminate the sequencing errors for pair-end sequencing data to provide much cleaner outputs, and consequently help to reduce the false-positive variants, especially for the low-frequency somatic mutations. While providing rich configurable options, AfterQC can detect and set all the options automatically and require no argument in most cases.

## Background

As next generation sequencing (NGS) technology being used more broadly in clinical applications, sequencing data quality control is becoming more important. In some NGS applications like ctDNA(circulating tumour DNA) sequencing [1], we need to detect ultra low frequency somatic mutations to help diagnosing cancers. However, the experiments (such like DNA amplification) and sequencing process always introduce errors and biases [2]. Typically the somatic mutation rate in ctDNA is near 1% for advanced tumour patients, and can be as low as 1% for early stage tumour patients [3], which is very close to the error rate of mainstream NGS platforms. The presence of these errors degrades the performance of variant calling tools in detection of true low frequency mutations while keeping false-positive mutations away. This problem drives us to not only apply better preprocessing with better quality control strategies and stricter filtering criterions, but also develop sequencing error profiling and correction algorithms to recognise and reduce errors as much as possible.

For sequencing data, some good tools can already perform quality control, such like FastQC [4] with per-base and per-sequence quality profiling functions and PRINSEQ [5] with FASTA/FASTQ statistics capability, while some other tools being able to read trimming, such like Trimmomatic [6] and SolexaQA [7]. Since the way to do data filtering depends on the QC result and the filtered data also need a post filtering QC, a tool with both rich QC and filtering functions is still wanted. Another improvement that can be made to these tools is overlapping analysis for pair-end sequencing, for which each DNA template is sequenced twice in forward and reverse directions. When the DNA template length is less than twice of the sequencing length, the pair of reads will be overlapped. Note that each base in the overlapping region is actually sequenced twice, so the inconsistency of these pairs may reflect the sequencing errors.

Another function needed for data preprocessing is cutting adapters. When the sequenced DNA template is shorter than sequencing length, part of sequencing adapters may be contained in the output reads. In this case, the adapters should be error-tolerantly detected and removed. Some tools like Trimmomatic [6] and Cutadapt [8] can handle such tasks, but they usually require users to input the sequence of the adapters, which are usually not well known for the people doing data analysis. By searching the best overlapping of each pair, AfterQC automatically detects and cuts adapters for pair-end data, with no need of adapter sequence input.

We will present AfterQC in this paper, a tool developed to address major practical sequencing data quality control and filtering problems. In addition to regular quality control functions like per-cycle base content and quality statistics, AfterQC also provides new functions like automatic trimming and overlapping analysis. For example, we found that some sequencers (like Illumina NextSeq series) may output a lot of polyX reads with high quality score. AfterQC can remove them using its polyX filter, while normal quality filters cannot. Another example is that we found if the amplification or sequencing process has serious strand bias, the sequence reads will show K-MER count bias (i.e. the counts of ATCGATCG and its reverse complement CGATCGAT are significantly different). Based on this finding, AfterQC provides K-MER counting based strand bias profiling. Another major contribution of this tool is overlapping analysis for pair-end sequencing data, which can be used to profile the sequencing error rate and apply error base correction or removing. For each input of a single or pair of FastQ files, AfterQC outputs a HTML report, which contains a quality control and data filtering summary, and a list of interactive figures.

## Methods

**Fig. 1:**
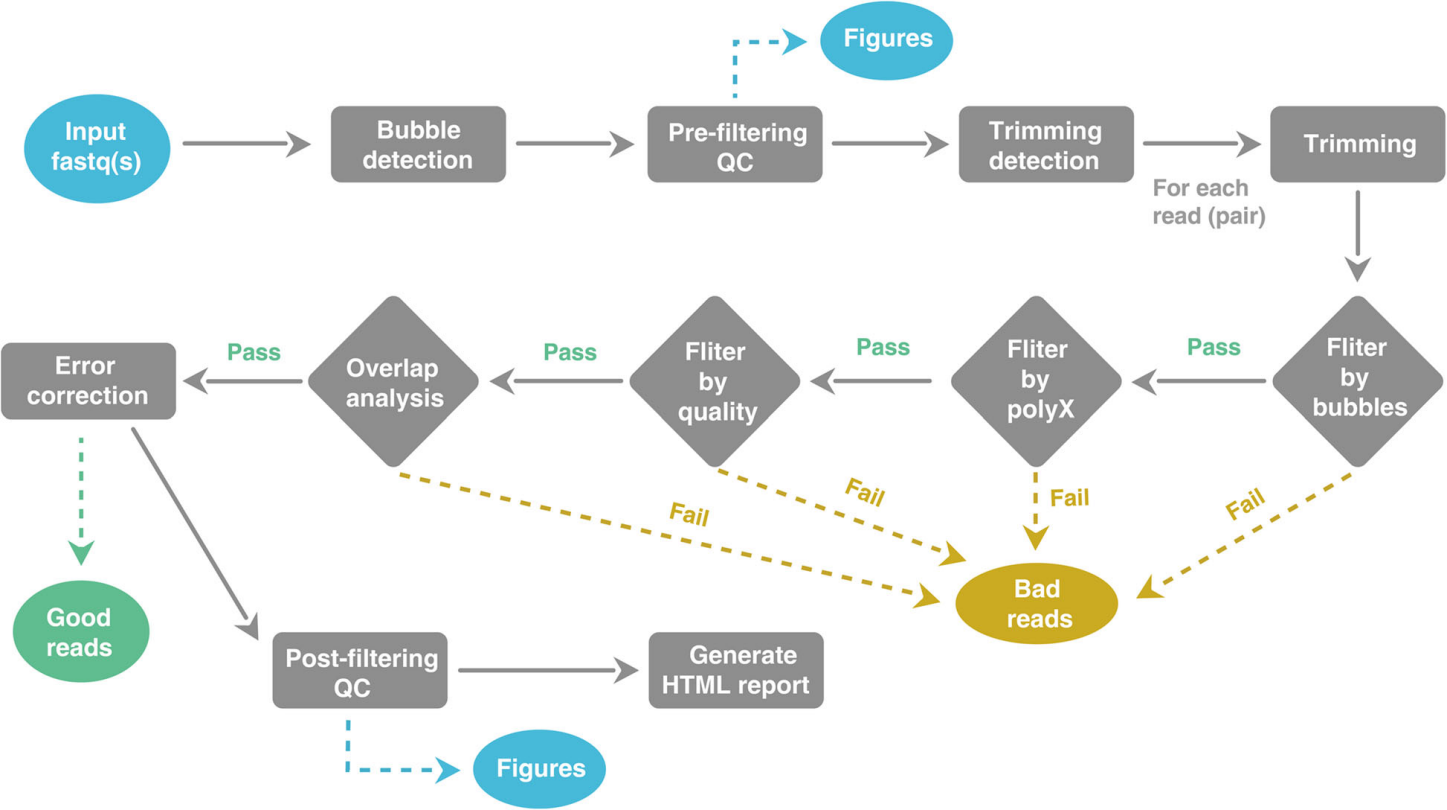
Pipeline diagram of AfterQC. For each single or pair of FastQ file(s), AfterQC will perform pre-filtering QC, automatic trimming, data filtering, error correction and post-filtering QC. Reads will be categorized as good or bad reads and stored separately, figures will be included in the final HTML report

AfterQC is designed to process FastQ files in batches. It goes through a folder with all FastQ files (can be single-end or pair-end output), which are typically data of a sequencing run for different samples, and passes each FastQ file or pair into the QC and filtering pipeline. As described in Fig. 1, firstly, AfterQC will run a bubble detection to find the bubbles raised during the sequencing process. Secondly, a pre-filtering QC will be conducted to profile the data with per-cycle base content and quality curves. Thirdly, AfterQC will do automatic read trimming based on data quality profiling. Fourthly, each read will be filtered by bubble filter, polyX filter, quality filter and overlapping analysis filters, the ones failed to pass these filters will be discarded as bad reads. Fifthly, an error correction based on overlapping analysis will be applied for pair-end sequencing data. Finally, AfterQC will store the good reads, perform post-filtering QC profiling and generate HTML reports.

### Bubble detection and visualisation

**Fig. 2:**
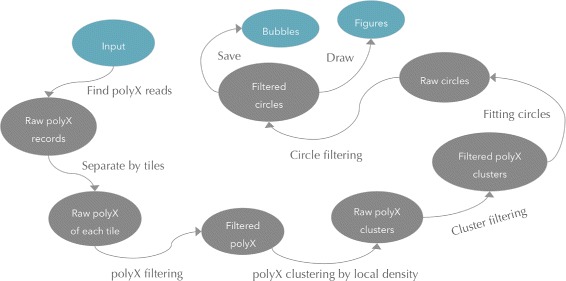
Algorithm diagram of deBubble. The major steps of this algorithm are polyX detection, polyX clustering and filtering, circle fitting and filtering

For Illumina sequencers, especially for those using two-channel SBS sequencing technology [9], we observed a phenomenon that more polyX reads could be found in the bubble areas than the background. Based on this phenomenon, we developed a method deBubble to visualise and detect bubbles. Firstly, we detect all polyX reads, separate them by tiles, and filter them by their local density since bubble areas tend to have higher polyX density. Secondly, we cluster the polyX reads into small sets, filter the clusters by features like size, shape and number of polyX reads. Thirdly, for each polyX cluster, we fit a circle to include all its polyX reads, and we also perform circle filtering to remove false positive bubbles. Finally, we plot the polyX and circle figures, and use these circles to filter out all the reads located in them. Figure 2 shows how we implement deBubble algorithm.

**Fig. 3:**
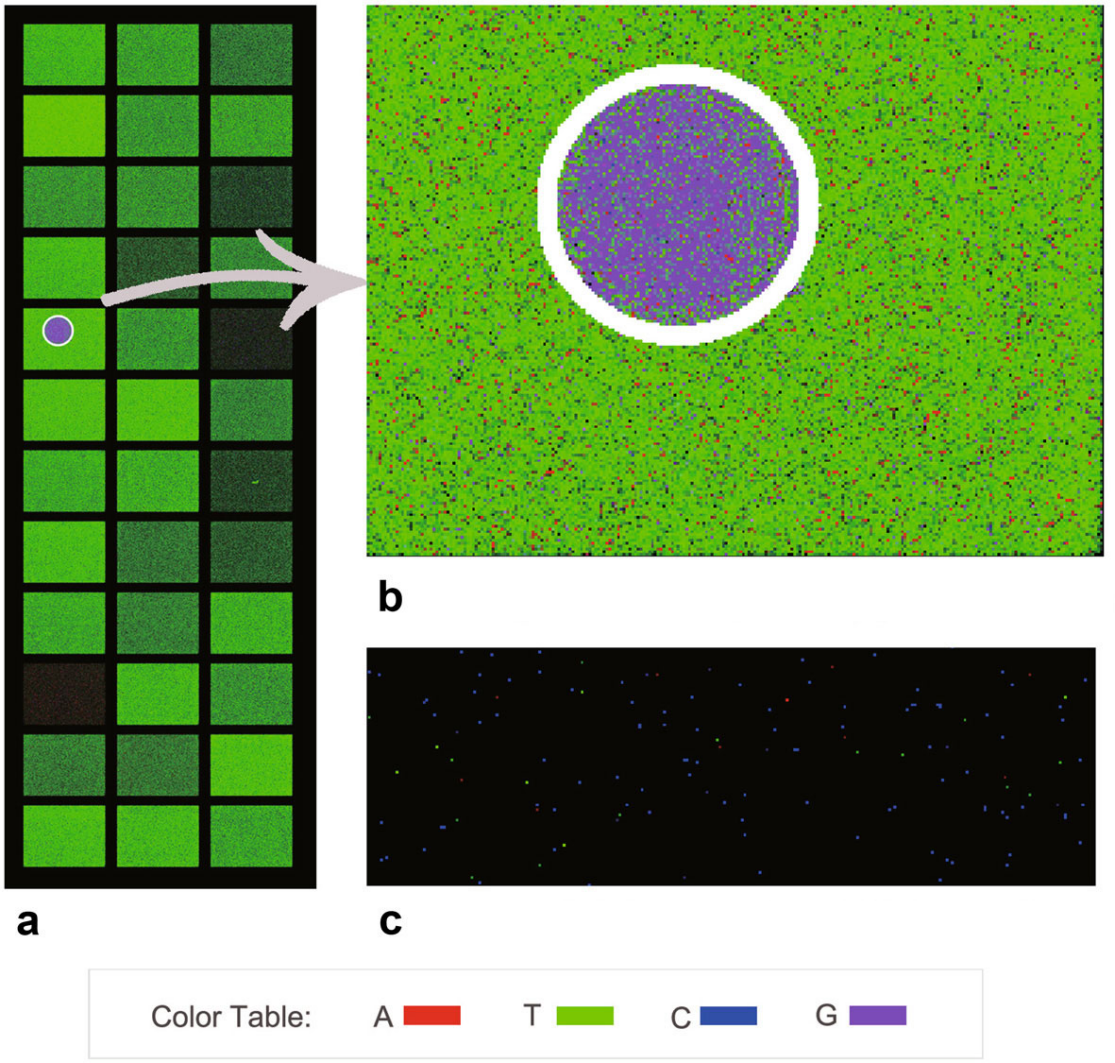
The output images of AfterQC deBubble. **a** is a sub-image of a lane of NextSeq 500 sequencer, from which we can find 1 bubble detected. **b** shows enlarged details of the bubble. **c** shows a sub-image of a tile of HiSeq 3000 with similar resolution of (**b**), which has much fewer polyX reads

Bubble detection is optional in AfterQC and is not enabled by default. According to our study, Illumina NextSeq sequencers are more likely to raise bubbles, so we suggest enabling this option for NextSeq sequencer outputs and disabling it for HiSeq sequencer outputs. Figure 3 shows a part of debubble’s output, from which we can also find that NextSeq sequencers produce much more polyX reads.

### Automatic trimming

In the whole sequencing process, the first several cycles can have more biases or errors since the signal coordination hasn’t been established yet, and the last several cycles can also have errors due to error accumulation and lack of following correction. In some cases, the beginning or ending of the reads may have significant statistical biases. For example, library preparation bias or sequencing bias can cause *GC* percentage higher than 70% at some beginning or ending cycles, and these cycles should be considered as abnormal cycles, and surely should be removed by some methods.

There are two strategies for trimming, namely local strategy and global strategy. Some tools, like Trimmomatic [6], apply local strategy, which perform trimming read by read. However local trimming has two drawbacks. The first drawback is that local trimming only uses the quality information for trimming, but cannot utilise the global statistical information to discover the abnormal cycles. The second drawback is local trimming results in unaligned trimming, which means duplicated reads may be trimmed differently, and consequently lead to failure of de-duplication tools like Picard [10]. Most of these de-duplication tools detect duplications only by clustering reads with same mapping positions.

**Fig. 4:**
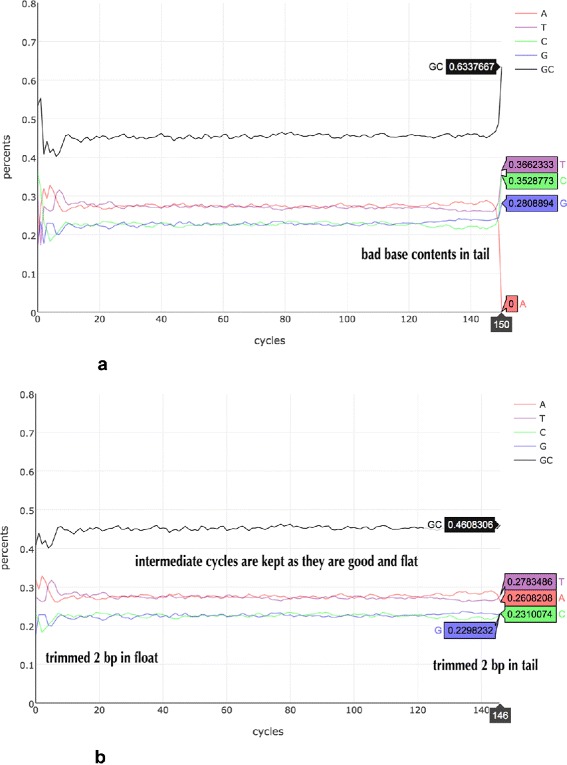
An example of how automatic trimming works. Data is obtained from a cell-free DNA quality control sample, and sequenced by Illumina NextSeq 500 sequencer. **a** is the base content percentage curve before trimming and filtering, from which we can find base contents change dramatically in front and tail; **b** is the curve after trimming and filtering, from which we can find that the bad cycles in the tail are all trimmed, while only part of the front is trimmed. This results from the fact that we use different thresholds for the front and tail, since unflatness in front is more probably caused by different fragmentation methods, while unflatness in tail is usually caused by lab preparation or sequencing artefacts

In contrast, AfterQC implements global trimming strategy, which means trimming all the reads identically. An algorithm is used to determine how many cycles to trim in the front and tail. The algorithm is based on such finding: the mean per-cycle base ratio curve is usually flat in the intermediate cycles, but may be fluctuant in the first and last several cycles. Also the intermediate cycles usually have higher mean quality score than the first and last cycles. Before trimming happens, AfterQC will do pre-filtering quality control to calculate the base content and quality curves. Our algorithm initialises the central cycle as a good cycle, and then expands the good region by scanning the base content and quality curves cycle by cycle, until it meets the front or end, or meet a cycle considered as abnormal. Then the cycles in the good region will be kept, and the rest cycles in the front and tail will be trimmed. Currently a cycle will be marked as abnormal if it meets at least one of following criteria: 1), too high or too low of mean base content percentages (i.e higher than 40%, or lower than 15%); 2), too significant change of mean base content percentages (i.e, ±10% change comparing to neighbour cycle); 3), too high or too low of mean GC percentages (i.e higher than 70%, or lower than 30%); 4), too low of mean quality (i.e. less then Q20). Figure 4 gives an example how automatic trimming works.

According to our experiments, AfterQC only trims very few cycles for data with good sequencing quality (i.e. 1 base in front, and 1 base in tail), so normally it will not significantly affect the data utilisation rate. However, for some extreme cases, the sequencing quality is quite low, and the mean base content percentage or quality curves can be totally chaotic. To not trim too many data for such cases, AfterQC limits the trimming cycles both in front and tail. The default setting is no more than 10% in front and no more than 5% in tail.

### Filtering

After trimming is done, AfterQC will apply a series of filters on the reads. AfterQC implements quality filters and polyX filters. Quality filters are trivial, which just count the number of low quality bases or N, calculate the mean quality of each read, and then determine whether to keep or discard this read. AfterQC implements an error-tolerantly method to detect polyX (X is one of A/T/C/G). Two arguments (*P* and *L*) are used to configure the polyX detection algorithm, *P* (default is 35) means how long the polyX sequence should have, while *L* (default is 2) refers to how many non-X bases can be tolerated in each polyX sub-sequence. According to our experiments, NextSeq sequencers are more likely to produce polyX reads, and most of them are polyG.

The order of applying different filters is not important. If one read is filtered out, a new sequence name containing the filter name will be assigned, and then this read will be streamed into the *bad* output.

### Overlapping analysis and error correction

Let *T* denote the length of a sequenced DNA template, and *S* denote the length of pair-end sequencing length, then the pair of reads will totally overlap if *T*≤*S*, will overlap with a length of 2*S*−*T*, if *S*<*T*<2*S*, and will not overlap if 2*S*≤*T*. Based on edit distance [11] optimisation, we developed a method to check how each pair of reads overlap, for data from pair-end sequencing. For a pair of reads *R*1 and *R*2, let *O* be the offset we place *R*2 under *R*1, then we’ll have vertically aligned subsequences *R*1_*o*_ and *R*2_*o*_, and we can calculate their edit distance *ed*(*R*1_*o*_,*R*2_*o*_). Our method optimises offset *O* to obtain the minimal edit distance, *ed*(*R*1_*o*−1_,*R*2_*o*−1_)<*ed*(*R*1_*o*_,*R*2_*o*_)<*ed*(*R*1_*o*+1_,*R*2_*o*+1_). We consider *R*1 and *R*2 overlapped at this offset *O* if this edit distance *ed*(*R*1_*o*_,*R*2_*o*_) and overlapped length *L*_*o*_ meet the thresholds.

**Fig. 5:**
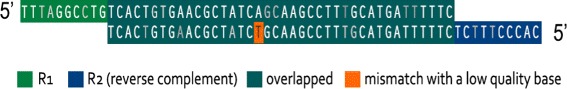
An example of overlapping analysis: the original DNA template is 60 bp long and sequencing length is 2×50, *R*1 and *R*2 have 40 bp overlap at offset 10, and the edit distance of the overlapped sub-sequences is 1. Brighter colour represents higher quality. A mismatch pair is found with high quality *A* and very low quality *T*, then *T* can be corrected

If a pair is overlapped, AfterQC will do overlapping analysis and error correction for it. If *ed*(*R*1_*o*_,*R*2_*o*_) is 0, it indicates no mismatch and no obvious sequencing error in the overlapped bases. Otherwise we should correct the overlapped mismatch or discard the reads if they cannot be corrected. For each pair with mismatch bases in overlapping region, we calculate the hamming distance *hd*(*R*1_*o*_,*R*2_*o*_) and check if it is identical to *ed*(*R*1_*o*_,*R*2_*o*_). If yes, it means there is only substitution difference between *R*1_*o*_ and *R*2_*o*_. For this case, we check the mismatch pairs to see if one base is of very high quality and the other is of very low quality. If it’s true, AfterQC will correct the low quality base according to its high quality mate. According to our results, most mismatch pairs have unbalanced quality scores. Figure 5 shows an example of overlapping analysis.

### Sequencing error profiling

As described above, AfterQC can detect the mismatches in the overlapping regions. For those reads with very long overlap (i.e. *overlap*_*len*>50), the edit distance of overlapped subsequences is mainly caused by sequencing errors, because an error-free overlapping is usually completely identical (edit distance should be 0). Based on this assumption, we can count the total bases and the mismatched bases in all overlapping regions. And we can consider the ratio of (*mismatch*/*total*) reflecting the sequencing error rate, which can be called estimated sequencing error rate. Furthermore, a mismatched pair usually consists of one high quality base (i.e. Q30+) and one low quality base (i.e. < Q15). In this case, we can consider that the low quality base in this pair is a sequencing error, and furtherly profile the sequencing error transform distribution (i.e. how many *T* bases are sequenced as *C*).

**Fig. 6:**
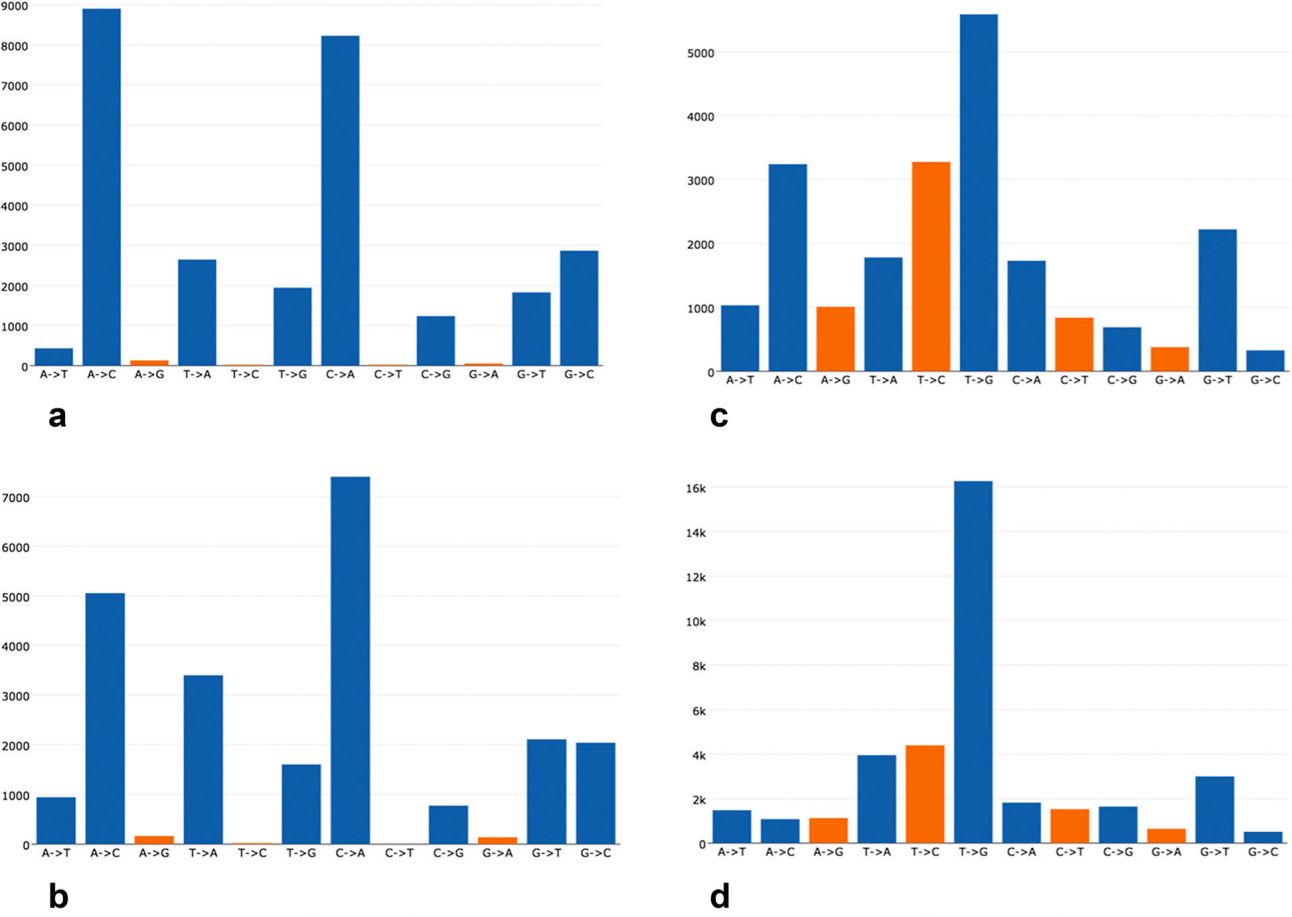
**a** Illumina NextSeq 500 output run #1. **b** Illumina NextSeq 500 output run #2. **c** Illumina HiSeq X-tenoutput run #1. **d** Illumina HiSeq X-tenoutput run #2. Sequencing error transform distribution is platform associated. Data are obtained from internal quality control DNA samples, and sequenced by Illumina HiSeq X10 sequencer and Illumina NextSeq 500 sequencer. Values in X-axis represent the sequencing error, and the values in Y-axis represent the counts calculated from a pair of FastQ files. Fig. (**a**) and (**b**) are profiled from two different sequencing runs from same NextSeq sequencers, while Fig. (**c**) and (**d**) are profiled from two different runs from different HiSeq sequencers. We can find that the patterns of **a**) and **b**) are nearly identical, while patterns of (**c**) and (**d**) are similar, but with noticeable difference

For each pair of pair-end sequenced FastQ files, AfterQC estimates such sequencing error rate and profiles the sequencing error transform distribution. By looking into the error distribution results from lots of sequencing data, we found an interesting phenomenon: error distribution is clearly sequencing platform dependent, different sequencing platforms have different error patterns, while the same sequencing platform’s different sequencing runs share similar patterns. Figure 6 shows an example of Illumina NextSeq sequencer patterns comparing with Illumina HiSeq sequencer patterns. An interesting phenomenon is that NextSeq sequencers produce very few *A*/*G* and *C*/*T* errors (the orange bars). We guess it is due to the two-colour system [9] adopted by NextSeq systems. In a Illumina two-colour system, base *A*, which requires both red and green light signals, is not easy to be mis-recognised as base *G*, which requires no light signals. Also base *C*, which requires only red light signal, can be clearly distinguished from base *T*, which requires green light signals.

### Automatic adapter cutting

**Fig. 7:**
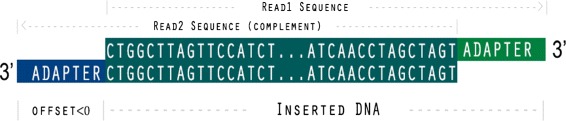
An example of automatic adapter detection and cutting. The offset makes the best alignment for this pair of reads is negative, which indicates that the length of inserted DNA is less than the sequencing length. When the offset is detected, it is trivial to calculate the overlapping region, and cut the adapter bases (outside overlapping region) from 3’ of both read1 and read2

When the DNA template length is less than the sequencing cycles, a part of 3’ adapter will be sequenced in the tail. From Fig. 7, we can see that when the inserted DNA template length *T* is less than sequencing length *S*, the offset *O* for the best overlapping will be negative. On the other hand, if we find that the optimal offset *O* for aligning the pair of reads is negative, we consider that the length of inserted DNA is smaller than sequencing length. Based on this rule, AfterQC implements automatic adapter cutting for pair-end sequencing data.

In the overlapping analysis process, we get the optimal offset *O* for the best local alignment of each pair. The overlap length of this pair can be directly calculated using the offset *O*. If *O* is found negative, the bases outside overlapping region will be considered as part of adapter sequences, and then be trimmed automatically.

### Quality profiling

**Fig. 8:**
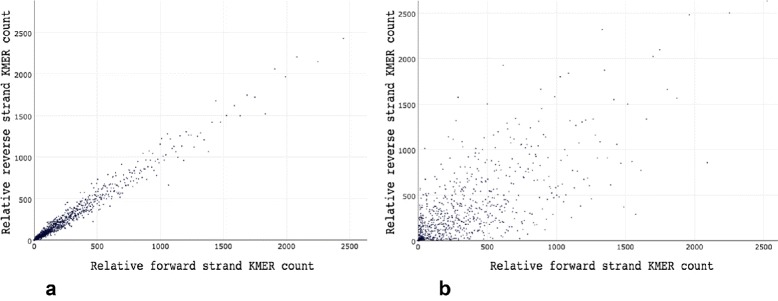
Two examples of strand bias profiling. X-axis is about the counts of relative forward strand K-MERs, while the Y-axis is about relative reverse ones. **a** shows a case of very little strand bias because most points are close to the line *y*=*x*, and (**b**) shows a case of serious strand bias because lots of points are close to X-axis and Y-axis, and repeat counts of some K-MERs are very high so the figure seems very sparse. Both files are downloaded from NCBI Sequence Read Archive (SRA), with accession numbers SRR1654347 and SRR2496735 [18]

Besides normal per-cycle base content and quality profiling, AfterQC implements two novel methods to give more information about sequencing quality: strand bias profiling to reflect amplification bias, and per-cycle discontinuity profiling to reflect sequencing quality instability. The first one is based on a hypothesis: if the DNA amplification process and sequencing process have only little non-uniformity, the repeat count of a short K-MER should be close to the repeat count of its reverse complement. So we plot each K-MER and its reverse complement’s counts, and check whether most points are near the line *y*=*x*. Figure 8 gives an example of K-MER based strand bias evaluation. The second method is based on another hypothesis: the mean discontinuity should be more or less stable for all sequencing cycles. For a short window of sequencing cycles, we use the average discontinued base number in this window to calculate the discontinuity. For example, *ATCGA* has a discontinued base number of 4 because all of the neighbour bases are different, while *AAAAA* has a discontinued base number of 0. If discontinuity drops down significantly cycle by cycle, it usually reflects a sequencing issue, which may be caused by the per-cycle washing process not working well.

### Software implementation

AfterQC can be viewed as a mix of quality control tools (i.e. FastQC) and data filtering/trimming tools (i.e. Trimmomatic, cutadapt). Table 1 gives a simple feature comparison of AfterQC with some existing tools. AfterQC differs from other tools by those features like overlapping analysis, bubble detection and automatic trimming. And for figure plotting, AfterQC switched from using matplotlib [12] to plotly.js [13] for creating interactive figures.

**Table 1:** A feature comparison of AfterQC with existing tools. From the table we can find that AfterQC is versatile on common quality control and data filtering tasks, and offers novel features not implemented by other tools before

	FastQC	Trimmomatic	Cutadapt	AfterQC
Quality Control	Rich functions	Few function	Few function	Rich functions
Auto Trimming	None	Read by read	Read by read	Global Trimming
Cutting adapter	None	Single-end/pair-end	Single-end/pair-end	Pair-end only
PolyX filtering	None	None	None	Supported
Figure plotting	Static	Static	Static	Interactive
Overlap analysis	None	Cutting adapter only	None	Supported with error correction
Sequence error profiling	None	None	None	Supported
Bubble detection	None	None	None	Supported
Programming Language	Java	Java	Python	Python, C
Speed	Fast	Fast	Fast	Fast only for single-end

Since AfterQC provides some functions that other high throughput sequencing QC or filtering tools do not possess, it usually runs slower than those other tools. In our evaluation, for pair-end sequencing data, AfterQC can process 2*240K pair-end reads per minute, while FastQC can process 2*1.5M reads per minute, which is 6X faster as AfterQC. However, the most time consuming parts of AfterQC are overlapping analysis and error correction processes, which are very useful for pair-end data. Actually, for single-end data, AfterQC can run as fast as FastQC, since no overlapping analysis is involved.

This tool is written in Python, with an edit distance module written in C. PyPy is supported for performance consideration. Currently, the fastest way to run AfterQC is using PyPy, but we are also re-implementing AfterQC using C/C++ only. The performance will be improved after the slow python code is replaced.

## Results and discussion

AfterQC has been used to process all of our 100+ runs’ sequencing data, most of which are cell-free DNA sequencing. According to previous studies, the mean length of cell-free DNA is around 167 bp [14]. This relatively short length of cell-free DNA makes AfterQC’s overlapping analysis very useful since most pairs of reads will be overlapped. The AfterQC results of our 100 runs’ data also confirm the reported length distribution. According to our results, sequencing quality can vary greatly with different runs, machines and samples. This suggests us to pay more attention to QC and data filtering, especially for clinical applications.

For pair-end sequencing, AfterQC provides an option to store only the overlapped sub-sequences, which means all pairs with no overlap, and outside overlapping areas will be discarded. Because the overlapped parts of each pair will be completely reverse complemented after overlapping analysis and error correction, this feature actually converts the pair-end sequencing data into high quality clean single-end sequencing data. Since most bases are double confirmed by pair-end sequencing, this overlapped data will have very high quality, and due to overlapping analysis based error correction, the sequence errors will be significantly eliminated.

**Fig. 9:**
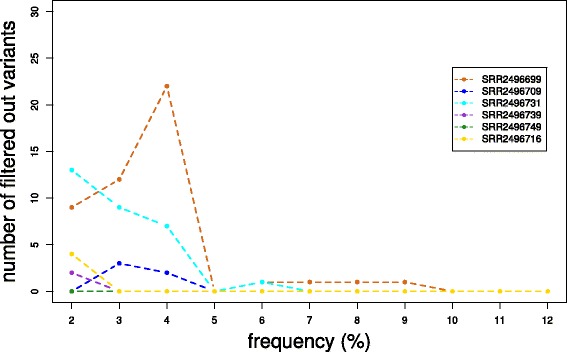
Six sample data were examined in this evaluation experiment, all of them were downloaded from NCBI Sequence Read Archive (accession numbers: SRR2496699 SRR2496709, SRR2496731, SRR2496739, SRR2496749, SRR2496716) [18]. AfterQC preprocessed every sample data and produced clean data files. BWA + Samtools + VarScan2 pipeline was applied on both raw data (not preprocessed) and clean data (AfterQC preprocessed). The variants called from raw data, but not called from clean data were counted. In this figure, values in X-axis denote the mutation frequency and the values in Y-axis denote the number of raw data only mutations, with frequency in each of the windows. Mutations with frequency lower than 2% are categorized to the first window. From this figure, we can learn that AfterQC helps filtering out lots of low frequency mutations, while seeing no difference for relatively high frequency (10%+) mutations

To evaluate how downstream analysis can benefit from AfterQC’s quality control, data filtering and error removing effort, we tested somatic variant calling pipelines with BWA [15] + Samtools [16] + VarScan2 [17] on both the raw data and AfterQC preprocessed data from several samples. From the experiment results, we found that large percentages of low-frequency somatic mutations called from raw data cannot be reproduced from the filtered clean data, especially for those mutations with frequency under 5%. This result indicates that a large percentage of low-frequency mutations may be false positives caused by errors, and AfterQC can help to remove them. Figure 9 gives an example showing that a large amount of low-frequency mutations are filtered out by AfterQC preprocessing.

## Conclusion

In summary, we developed a tool called AfterQC with rich quality control, data filtering, error profiling and correction functions for next generation sequencing data. AfterQC is fully tested with a large amount of data and has been accepted by some community users. The overlapping analysis and other techniques used in this tool make it possible to generate high quality clean reads, and make it very useful for low frequency somatic mutation detection in deep sequencing applications.

## References

[1] Schwarzenbach H, Hoon DS, Pantel K (2011). Cell-free nucleic acids as biomarkers in cancer patients. Nat Rev Cancer.

[2] Quail MA, Smith M, Coupland P, Otto TD, Harris SR, Connor TR, Bertoni A, Swerdlow HP, Gu Y (2012). A tale of three next generation sequencing platforms: comparison of ion torrent, pacific biosciences and illumina miseq sequencers. BMC genomics.

[3] Newman AM, Bratman SV, To J, Wynne JF, Eclov NC, Modlin LA, Liu CL, Neal JW, Wakelee HA, Merritt RE, Shrager JB (2014). An ultrasensitive method for quantitating circulating tumor dna with broad patient coverage. Nature medicine.

[4] Andrews S. A Quality Control Tool for High Throughput Sequence Data. http://www.bioinformatics.bbsrc.ac.uk/projects/fastqc/. Accessed 7 Dec 2016.

[5] Schmieder R, Edwards R (2014). Quality control and preprocessing of metagenomic datasets. Bioinformatics.

[6] Bolger AM, Lohse M, Usadel B (2014). Trimmomatic: A flexible trimmer for illumina sequence data. Bioinformatics.

[7] Cox MP, Peterson DA, J BP (2010). Solexaqa: At-a-glance quality assessment of illumina second-generation sequencing data. BMC Bioinforma.

[8] M M (2011). Cutadapt removes adapter sequences from high-throughput sequencing reads. EMBnet J.

[9] Illumina: Two-Channel SBS Sequencing Technology. San Francisco; 2015. https://www.illumina.com/content/dam/illumina-marketing/documents/products/techspotlights/techspotlight_two-channel_sbs.pdf.

[10] Institute B. A Set of Java Command Line Tools for Manipulating High-throughput Sequencing (HTS) Data and Formats. https://github.com/broadinstitute/picard. Accessed 7 Dec 2016.

[11] Gao X, Xiao B, Tao D, Li X (2010). A survey of graph edit distance. Pattern Anal Applic.

[12] Hunter JD (2007). Matplotlib: A 2d graphics environment. Comput Sci Eng.

[13] Sievert C, Parmer C, Hocking T, Chamberlain S, Ram K, Corvellec M, Despouy P. MCreate Interactive Web Graphics Via Plotly.js. https://github.com/plotly/plotly.js. Accessed 7 Dec 2016.

[14] Snyder MW, Kircher M, Hill AJ, Daza RM, Shendure J (2016). Cell-free dna comprises an in vivo nucleosome footprint that informs its tissues-of-origin. Cell.

[15] Li H, Durbin R (2009). Fast and accurate short read alignment with burrows–wheeler transform. Bioinformatics.

[16] Li H, Handsaker B, Wysoker A, Fennell T, Ruan J, Homer N, Marth G, Abecasis G, Durbin R (2009). The sequence alignment/map format and samtools. Bioinformatics.

[17] Koboldt DC, Zhang Q, Larson DE, Shen D, McLellan MD, Lin L, Miller CA, Mardis ER, Ding L, Wilson RK (2012). Varscan 2: somatic mutation and copy number alteration discovery in cancer by exome sequencing. Genome research.

[18] Hyman D, Solit D, Arcila M, Cheng D, Sabbatini P, Baselga J, Berger M, Ladanyi M (2015). Precision medicine at memorial sloan kettering cancer center: clinical next-generation sequencing enabling next-generation targeted therapy trials. Drug discovery todal.

